# Decrease in Stroke Diagnoses During the COVID-19 Pandemic: Where Did All Our Stroke Patients Go?

**DOI:** 10.2196/21608

**Published:** 2020-10-21

**Authors:** Adrienne Nicole Dula, Gretchel Gealogo Brown, Aarushi Aggarwal, Kal L Clark

**Affiliations:** 1 Department of Neurology Dell Medical School The University of Texas at Austin Austin, TX United States; 2 Department of Diagnostic Medicine Dell Medical School The University of Texas at Austin Austin, TX United States; 3 School of Nursing University of Texas Health Science Center at San Antonio San Antonio, TX United States; 4 Long School of Medicine University of Texas Health San Antonio, TX United States; 5 Department of Radiology University of Texas Health Science Center at San Antonio San Antonio, TX United States

**Keywords:** stroke, ischemic stroke, COVID-19, SARS-CoV-2, emergency medicine, cerebrovascular

## Abstract

Despite the evidence suggesting a high rate of cerebrovascular complications in patients with SARS-CoV-2, reports have indicated decreasing rates of new ischemic stroke diagnoses during the COVID-19 pandemic. The observed decrease in emergency department (ED) visits is unsurprising during this major crisis, as patients are likely to prioritize avoiding exposure to SARS-CoV-2 over addressing what they may perceive as mild symptoms of headache, lethargy, difficulty speaking, and numbness. In the central and south Texas regions where we practice, we suspect that patient admission, treatment, and discharge volumes for acute stroke treatment have decreased significantly since COVID-19–related shelter-at-home orders were issued. Symptoms of stroke are frequently noticed by a family member, friend, or community member before they are recognized by the patients themselves, and these symptoms may be going unnoticed due to limited face-to-face encounters. This possibility emphasizes the importance of patient education regarding stroke warning signs and symptoms during the current period of isolation and social-distancing. The south Texas population, already saddled with above-average rates of cardiovascular and cerebrovascular disease, has a higher stroke mortality rate compared to Texas and U.S. averages; however, the number of patients presenting to EDs with acute ischemic stroke diagnoses is lower than average. In our viewpoint, we aim to present the relative literature to date and outline our ongoing analyses of the highly affected and diverse stroke populations in San Antonio and Austin, Texas, to answer a simple question: where did all our stroke patients go?

## Introduction

In the midst of a pandemic, the advice from medical professionals to “stay home, save lives” may be preventing patients from seeking medical care when symptoms of stroke arise due to fear of contracting COVID-19. Anecdotal reports across the United States are highlighting missed care during the current COVID-19 pandemic [1-3]; the World Health Organization has acknowledged a marked decrease in stroke presentations and a widespread impact of the pandemic on stroke care [4]. Local and state shelter-at-home orders have imposed strict limitations on clinic and hospital access, restrictions on nonessential travel, social distancing policies, and mandated isolation of populations that are especially vulnerable to COVID-19 infection, such as older and immunocompromised people. In our persistent efforts to educate communities about COVID-19 infection risk, we may be undermining potentially lifesaving public health education campaigns for emergent medical conditions such as heart attack or stroke. The aim of this viewpoint is to present the relative literature to date and outline ongoing analyses of the highly affected and diverse stroke populations in San Antonio and Austin, Texas.

In the central and south Texas regions where we practice, we suspect that patient admission, treatment, and discharge volumes for acute stroke treatment have decreased significantly since COVID-19–related shelter-at-home orders were issued. This decrease appears to mirror sharp downward trends in emergency department (ED) visits since Texas municipalities began enforcing local guidelines for social distancing. This is especially concerning for the San Antonio metropolitan statistical area. Our population is already saddled with above-average rates of cardiovascular and cerebrovascular disease and has a higher stroke mortality rate compared to the Texas and US averages [5-7]. Hesitation to leave shelter-in-place due to COVID-19 and present to the ED could result in worse outcomes, particularly for ischemic stroke [8,9]. To put it simply: where did all our stroke patients go?

Answering this question presents a unique opportunity to maximize the information-laden infrastructure already in place for tracking acute stroke care: “Get With The Guidelines” (GWTG) stroke registry of the American Heart Association/American Stroke Association (AHA/ASA). Required for Joint Commission and Det Norske Veritas Germanischer Lloyd (DNV-GL) stroke center certification, GWTG registries provide a mechanism for hospitals to track the efficiency and effectiveness of their acute stroke care for continuous program evaluation and quality improvement. It should be noted that in April 2020, a COVID-19 item for tracking testing and outcomes was added to the registry case report form.

## Increasing Stress on the Hospital System

Without the typical rigorous process of development, refinement, and peer review of national recommendations, on March 20, 2020, the AHA/ASA released a broad but flexible policy statement that reflects both the commonality of the pandemic across the United States and the individual variability at local sites [10]. The report was issued as a temporary statement and interim stopgap opinion pending a more thorough and contemplative process. Obstacles to acute stroke care were cited, including shortages in personal protective equipment (PPE), hospital personnel, and hospital beds.

Initial reports from other countries have already highlighted the stress of COVID-19 on their intensive care units (ICUs) and resources [11-13]. A recent case series of critically ill patients with COVID-19 in Seattle, Washington, reported a median ICU stay of 14 days and a median duration of mechanical ventilation of 10 days [14]. With EDs and ICU triaging and caring for increasing numbers of patients with COVID-19, the COVID-19 pandemic will have a tremendous impact on available resources for the triage and treatment of acute ischemic stroke.

Many patients with stroke often fail to recognize mild symptoms such as visual field disturbance, facial droop, or neglected extremities. Symptoms of stroke are frequently noticed by a family member, friend, or community member before they are recognized by the patients themselves, which emphasizes the importance of patient education regarding warning signs and symptoms of stroke during the current exigent period of isolation and social distancing.

## Suggested Guidelines for Stroke Care

Current practice for the management of acute ischemic stroke will require modification, and although it has been recommended that guidelines should be relaxed while maintaining a high standard quality of care [15], most published guidelines or recommendations (Table S1, Multimedia Appendix 1) state that established guidelines should be followed [10,12,14,16-22]. Others have published suggestions for acute ischemic stroke care with modifications to standard treatment guidelines [23-25]. There is an overall collective aim to avoid contributing to the rapid spread of COVID-19 while conserving what are likely to be very limited resources (including personnel, ICU and hospital beds, and physicians) while providing acute ischemic stroke care [15,23]. The concept of a “protected code stroke” during a pandemic, as in the case of COVID-19, was introduced for Canadian practice by Khosravani et al [20]; this concept provides a framework for key considerations, including screening, PPE, and crisis resource management.

Recommendations covering the areas of education, screening, imaging, treatment, transfer, discharge, and follow-up procedures have been published. A number of publications have highlighted the importance of improving educational outreach for health professionals and the public, particularly those at high risk of stroke, to recognize stroke and call emergency medical services (EMS), thus avoiding significant delays and worse outcomes [12,13,19,26,27]. To minimize risk of infection, many guidelines suggest screening for COVID-19 symptoms and exposure as soon as possible, including remotely or by EMS, and communicating the results to the stroke team [14,16,18,20,22,25].

Recommended strategies regarding imaging include establishing a COVID-specific scanner [16,17] and performing magnetic resonance imaging (MRI) first so that patients do not require multiple scanning sessions [25]. Identification of transient ischemic attacks (TIAs) and mild strokes without deficits and no indication for emergent treatment could be addressed with remote management [14,25,26]. While most published recommendations indicate that established guidelines should be followed regarding treatment, French interventionalists suggest withholding treatment from patients in the ICU who test positive for COVID-19 [22] and others suggest remote review of treatment eligibility, including functional exams [10,25]. In specific reference to endovascular therapy (EVT) procedures, some guidelines recommend early intubation of patients prior to initiation [20,21,28] or require confirmed COVID-19–negative status prior to the procedure [24]. Rapid discharge of patients who can be managed at home [12,14,17,25] and remote follow-up consultations [12,14,17,19,20,22,27] are also mentioned as strategies. Finally, early recognition of the need for transport to a designated stroke center can reduce interfacility transfers, effectuating a reduction in potential infectious exposure [26].

## Decrease in Ischemic Stroke Patients

As people continue to adjust to social distancing, a shift in the epidemiology of stroke and other medical conditions will most likely be observed, as is being seen in myocardial infarction [29] and in other countries [30-37] in the context of acute ischemic stroke. Although there is evidence to suggest a high rate of cerebrovascular complications in patients with SARS-CoV-2 infection [38-43], anecdotal reports indicate a falling rate of new ischemic stroke admissions [30-32,34,37,44-48], stroke code activations [30,36,49-53], imaging numbers [44,45], and diagnoses [45].

This decrease in patient load is hypothesized by some to be driven by fewer patients presenting to the ED [54,55]. Studies have also noted a decrease in admissions of TIAs [31] and a decrease in patients presenting with mild symptoms, as demonstrated by higher National Institutes of Health Stroke Scale (NIHSS) scores [32,34,50] and decreased proportion of large vessel occlusions (LVOs) [45]. These data may indicate that a smaller proportion of patients are seeking services for mild symptoms [45].

Additionally, decreases in acute ischemic stroke treatment numbers have been noted for both thrombolysis [46,53,56,57] and EVT [30,33,47,53,56], with an excellent meta-analysis presented by July et al [53], including data from nine studies [30,56,58-64]. Details of each of these studies, as well as other related studies [65,66], can be found in Table S2 (Multimedia Appendix 1). Interestingly, despite noted decreases in EVT numbers overall, some studies have shown that the proportion of patients receiving EVT increased [48,53], possibly due to an increase in the number of late presenters [34,58] or an increase in the number of patients eligible for EVT. Upon examination, the majority of studies reported no change in process times, such as time last known well (TLKW) to arrival [45,47,49,51], TLKW to imaging [45], TLKW to thrombolysis [45,48,49,51], and length of hospital stay [47]. However, other studies reported an increase in the process time from TLKW to EVT procedure [33] or no change in the length of hospital stay [47].

Of particular interest for our planned analyses is the report by Kansagra et al [44] quantifying the stroke imaging load for each state via the RAPID software database (iSchemaView Inc). Texas-specific data show that prepandemic (February 1 through February 29, 2020), Texas had a mean of 63.3 patients per day (95% CI 60.2-66.5), and in the early pandemic (March 26 through April 8, 2020), the mean was 43.1 patients per day (95% CI 40.1-46.2), representing a change of –31.8% (95% CI –25.8% to –37.6%). There were no significant differences with respect to age, sex, race, vascular risk factors, or severity. Upon examining stroke type, the proportion of new LVOs nearly doubled in the COVID-19 period (n=20, 38%, vs n=59, 21%, *P*=.01) relative to the pre-pandemic timeframe. Despite differences in proportions, the mean number of LVO patients in the COVID-19 period (0.43 per day) did not differ significantly (*P*=.61) from that pre–COVID-19 (0.39 per day). Finally, evaluation of time revealed that patients treated during the COVID-19 period had no significant delay from TLKW to arrival or from arrival to imaging or treatment [45]. These results further support that the driving factor for the decrease in stroke volume is that patients experiencing mild strokes are not seeking acute care.

Accurately identifying the root cause of the decrease in stroke volume is challenging due to a combination of socioeconomic and pathophysiologic factors. Widely observed decreases in acute ischemic stroke presentation are potentially influenced by the risk profile of the population: during the COVID-19 era, patients with risk factors such as hypertension, hyperlipidemia, coronary artery disease, lack of insurance, or urban location increased their proportion of the stroke cohort [52]. Population health strategies to reduce COVID-19 may also lower infection rates with vasculotropic viruses and allergens that can trigger atherosclerosis and plaque rupture, which may result in neurovascular and cardiovascular morbidity [67,68].

## Disparities

There is increasing evidence that some racial and ethnic minority groups are being disproportionately affected by COVID-19 [69-73]. Hypothesized mechanisms include an elevated severity of response to SARS-CoV-2 and increased socioeconomic risk [74]. Persons who are African American, Black, or Latino are contracting SARS-CoV-2 at higher rates and experiencing higher mortality [75-78], and comorbidities may explain these differences [74]. Due to this increased risk, Black and Hispanic or Latino patients, particularly those without health insurance [52], may avoid medical care. Observations from a telestroke registry in North Carolina reported that a lower percentage of Black patients presented during the pandemic (13.9% versus 29% before the pandemic, *P*<.001).

Racial and ethnic minority groups who are more likely to rely on the ED for primary care may avoid seeking primary care because of concerns about the infection risk in the ED. The avoidance of primary care for chronic disease management can subsequently manifest in an increase in emergent hospitalization for stroke. A statistically significant increase in the proportion of Black and Hispanic patients presenting with strokes was noted in California, Pacific hospitals, Western hospitals, and all hospitals in the United States during various months studied, comparing 2020 to 2019 [79]. It is not clear what proportion of patients with severe strokes are foregoing medical care or are otherwise underdiagnosed.

Further work is needed to explore the complex interplay of socioeconomic factors and pathophysiologic mechanisms and the impact this interplay has on acute stroke presentation in minority populations. Until this work is performed, it may be difficult to effectively target population health resources and address the disparity.

## Planned Analyses

Unfortunately, when patients misinterpret stroke symptoms or assume that the symptoms will resolve without intervention, they are delaying clinical care. Without emergency medical attention, strokes can cause devastating and irreversible damage, with the extent being largely dependent on the timing of the intervention. Furthermore, infection prevention protocols may complicate discussions between the patient and emergency responders as well as subsequent management and treatment. Hospital and EMS procedures are consistently adapting to the situation as reports from around the world detail experiences and provide data on which to base acute stroke care decisions during this pandemic.

It is necessary to comprehensively evaluate the impact of current stay-at-home orders and patient fears on incidence and severity of ischemic stroke. Metrics can be extracted from the GWTG stroke registry data along with related neuroimaging exams from hospitals in Texas metropolitan areas (San Antonio and Austin). Unlike San Antonio, Austin and its surrounding counties have lower cardiovascular and cerebrovascular disease rates and stroke mortality rates compared to Texas and US averages due to a combination of socioeconomic status, public health, infrastructure, and demographic differences. Thus, Austin will serve as a de facto control group. For this study, which has been approved by the University of Texas Health Science Center and The University of Texas at Austin Institutional Review Boards, data from San Antonio and Austin will be divided into three cohorts: 

1. Prior to the COVID-19 pandemic: Records of stroke patients with admission dates 15 months prior and up to March 31, 2020, when the governor of Texas issued Executive Order No. GA-14, which effectively limited out-of-household social gatherings and in-person contact to only those necessary for providing or obtaining essential services.
2. Stay-at-home order in effect: Records of stroke patients with admission dates from April 1 to April 30, 2020, while the above executive order was in effect.
3. Stay-at-home order rescinded: Records of stroke patients with admission dates up to 15 months after May 1, 2020, when the above executive order was discontinued.

A fourth cohort of stroke patients will be created if stay-at-home orders are reinstated during the 15-month period after May 1, 2020. We will test the hypothesis that the overall stroke presentation rate (including positive and negative cases), absolute numbers of stroke diagnoses, and stroke interventions decline during COVID-19 stay-at-home orders are in effect compared to control time epochs. We plan to analyze admission, treatment, and discharge variables within and between time cohorts and metropolitan areas, and when available, we will analyze data on COVID-19 testing and outcomes that occurred during stroke treatment. San Antonio and Austin-area GWTG data to be extracted for the study data set will include patient demographics (age, gender, race/ethnicity)

- Arrival and admission data (locations where stroke symptoms were discovered, mode of arrival to ED, vital signs, laboratory test results, height/weight/BMI, inpatient unit assigned for stroke care)
- Medical history (pertinent medical history and medications)
- Diagnosis and evaluation (initial NIHSS score and exam findings)
- Symptom timeline (date/time of last known well, date/time of symptom discovery)
- Brain imaging (imaging modality, initiation date/time, interpretation of findings)
- Acute therapeutic interventions (intravenous thrombolytic therapy, EVT, related complications)
- Other in-hospital treatment and screening (interventions for venous thromboembolism, anticoagulation, bacterial/viral infection)
- Discharge status (modified Rankin Scale score at discharge, ambulatory status)
- Discharge treatment (antithrombotic therapy, antihypertensive therapy, statin therapy, antihyperglycemic therapy, patient education, follow-up diagnostic tests and procedures)

We anticipate a sample of n=1000 GWTG records from 11 hospitals (of which 4 are comprehensive stroke centers) in the San Antonio and Austin metropolitan statistical areas for the study data set. Kruskal-Wallis and Levene tests will be used to assess differences in categorical and continuous GWTG variables between and within time periods and metropolitan area cohorts, and segmented regression will be used to assess whether stroke presentation, interventions, and outcomes are more or less severe over the pre-, during, and post–COVID-19 stay-at-home order time periods.

## Conclusions

Fear and heightened caution are not the only factors that contribute to reduced ED visits during burdensome times. Looking retrospectively at ED trends during natural disasters offers another perspective on decreasing ED visits. A study that analyzed the effects of Hurricane Sandy, a hurricane that struck the east coast of the United States in 2012, on cardiovascular events found that in the days following the disaster, there were increases in incidence and mortality of myocardial infarction and in stroke incidence [80]. The authors referenced a multitude of studies documenting increased incidence of cerebrovascular events during natural disasters or severe weather events. Although the underlying etiologies of that increase have not been proven, possibilities include emotional stress related to increased platelet activation [81] and delayed treatment due to disrupted transportation networks, health care capacity, and supply chains. In terms of cerebrovascular events, the COVID-19 crisis may represent the worst-case scenario: fear of nosocomial infection causing health care avoidance when, for physiological and/or psychological reasons, the population is most vulnerable.

An observed decrease in ED visits should come as no shock during times of major crises, as patients prioritize safety from the “current danger” over what they may perceive as mild symptoms of chest pain, abdominal pain, weakness, etc., that they hope will “resolve on their own.” This trend of decreased ED use was previously observed during the 2003 severe acute respiratory disorder (SARS) outbreak, in which ED visits at the height of the epidemic in Taipei showed a 51.6% decline, with a mean of 115.4 visits (SD 16.7) compared to the previous year (mean 238.3 visits, SD 33.4, 95% CI of the mean difference 109.4-136.3; *P*<.01) [82]. Further analysis showed persistence of this pattern for the 2015 Middle East respiratory syndrome (MERS) outbreak in South Korea as well. The age-standardized prevalence ratio for ED visits per 100,000 people in June 2015 decreased to 0.72, with June 2014 and 2016 as references. The mean age-standardized prevalence for June 2015 was 708.48 (95% CI 706.15-710.82), and the mean number of ED visits for ischemic stroke in 2015 (5406) decreased by 33.1% in comparison to that of the year before the outbreak (6185 in June 2014) and the year after the outbreak (6776 in June 2016) [83]. Subgroup analyses in that study identified a concerning phenomenon: the volumes of high-acuity diseases such as myocardial infarction and ischemic stroke demonstrated a significant decrease in ED visits (14.0% and 16.6% decreases, respectively). These historical trends, including those observed for the current COVID-19 pandemic, raise the hypothesis that fear of nosocomial infection transmission can have deleterious population health consequences.

## Appendix

Multimedia Appendix 1 Suggested guidelines and protocols for management of acute stroke and key studies evaluating acute stroke trends during the COVID-19 pandemic.

## Abbreviations

AHA/ASA: American Heart Association/American Stroke Association
DNV-GL: Det Norske Veritas Germanischer Lloyd
ED: emergency department
EMS: emergency medical services
EVT: endovascular therapy
GWTG: Get With The Guidelines
ICU: intensive care unit
LVO: large vessel occlusion
MERS: Middle East respiratory syndrome
NIHSS: National Institutes of Health Stroke Scale
SARS: severe acute respiratory syndrome
TIA: transient ischemic attack
TLKW: time last known well
