# Supplementary material for: Decrease in Stroke Diagnoses During the COVID-19 Pandemic: Where Did All Our Stroke Patients Go?
Source: JMIR Aging. 2020 Oct 21;3(2):e21608. doi: 10.2196/21608 (PMC7581311; doi:10.2196/21608)
Supplement: Multimedia Appendix 1 [file aging_v3i2e21608_app1.docx]

Table S1. Suggested guidelines and protocols for management of acute stroke during the COVID-19 pandemic.

| Guideline group and reference | Guideline name | Date of update | Recommendations |
| --- | --- | --- | --- |
| Société Française Neuro-Vasculaire and Société Française de Neuroradiologie [22] | Recommendations of the Société Française Neuro-Vasculaire and Société Française de Neuroradiologie for the Care of Patients  With Stroke During the COVID-19 Coronavirus Epidemic | March 1, 2020 | Screening: Risk of COVID-19 assessed as soon as possible  Testing: Perform COVID-19 testing on patients with symptoms, otherwise send to neurovascular unit  Care Coordination: Management by the neurovascular team according to current recommendations  Thrombolysis/EVT^a^: Do not offer invasive treatment for COVID-19–positive patients already in intensive care  Contact Minimization: Patients with symptoms must wear a mask and personnel must wear PPE^b^  Face-to-face follow-up consultations are postponed  Approach to COVID-19–positive patients: Admitted to dedicated unit (when available) or COVID-19 sector of the Neurological Unit and monitored by the neurological team |
| Royal College of Physicians [25] | Clinical guide for the management of stroke  patients during the coronavirus pandemic | March 23, 2020 | Screening: Risk of COVID-19 assessed as soon as possible. This may be face-to-face with PPE, but only one stroke specialist to consider, with secondary reviews performed via video or telephone  Imaging: Consider first-line MRI^c^  Consider CTA^d^ for all patients with carotid territory symptoms  Rapid introduction of artificial intelligence to speed up imaging  Remote interpretation; adoption should be coordinated across networks  Care Coordination: Virtual consultant cover to facilitate shadow rotations  Thrombolysis/EVT: Remote telemedicine review of all potential thrombolysis patients  Contact Minimization: Rapid discharge to Early Supported Discharge Services to minimize time spent is hospital  Telephone triage for all TIA^e^ referrals followed by virtual clinic |
| Stroke Unit and Neurosonology Laboratory, Padua University Hospital, Padua, Italy [16] | Acute Stroke Management Pathway During Coronavirus-19 Pandemic | March 25, 2020 | Screening: Risk of COVID-19 assessed as soon as possible  Imaging: COVID-19–positive and suspected COVID-19: cerebral CT^f^, CTA, and CTP^g^ on mobile CT outside the ED^h^  Thrombolysis/EVT: Standard thrombolysis protocol and EVT in angio-suite with PPE  Contact Minimization: Patients and health care personnel must wear recommended PPE  Approach to COVID-19–positive patients: Patients will be transferred to Infectious Disease ward and monitored by stroke team |
| Sunnybrook Health Sciences Centre, University of Toronto, Canada [20] | Protected Code Stroke  Hyperacute Stroke Management During the Coronavirus Disease 2019 (COVID-19) Pandemic | March 26, 2020 | Screening: Risk of COVID-19 assessed as soon as possible by paramedics; should include an infection control screen and travel history screen  Thrombolysis/EVT: Local and regional practice recommendations should always be followed  Intubate early for increasing O_2_ requirements  Contact Minimization: Routine code stroke, contact, and droplet precautions should be used  Placement of a surgical mask on the nonintubated patient after securing PPE for all team members  Reduce frequency of assessments to what is most essential |
| American Heart Association/American Stroke Association Stroke Council Leadership [10] | Temporary Emergency Guidance to US Stroke Centers  During the Coronavirus Disease 2019 (COVID-19) Pandemic | March 31, 2020 | Care Coordination: Minimize the use of scarce PPE  Maintain multispecialty and collaborative effort  Fewest possible team members to see patients with code stroke and in rooms for follow-up visits  Thrombolysis/EVT: Full adherence to guidelines may be challenging; however, needed treatment should be offered to the fullest extent possible  Contact Minimization: Telemedicine for NIHSS^i^  Follow protocols for health and safety (hand washing, PPE, COVID-19 testing, and self-quarantine) |
| Editorial (Minhang Hospital, Fudan University, Shanghai, China; Kings College London, United Kingdom; Perelman School of Medicine at the University of Pennsylvania, Philadelphia) [13] | Challenges and Potential Solutions of Stroke  Care During the Coronavirus Disease 2019 (COVID-19) Outbreak | March 31, 2020 | Care Coordination: Establishment of stroke networks and care systems able to deliver high-quality emergency stroke care at all times  Establishment of centralized stroke treatment centers  Outreach: Inform the EMS^j^ and the public that stroke centers are safe and fully operational even during crises  Improve education of health professionals and the public, especially those who are at high risk of stroke, to recognize stroke and call EMS immediately |
| Italian Stroke Organization [24] | Stroke Care During the COVID-19 Pandemic | April 1, 2020 | Screening: Remote FAST^k^ scale screen for COVID-19 symptoms  Pretriage, screen again for symptoms or exposure  Imaging: Recommend system respiratory imaging  Thrombolysis/EVT: Recommend starting thrombolysis then performing COVID-19 test  EVT candidate, wait for results of swab and if needed, treat patient in dedicated COVID-19 area  Hospitalization: Different locations/pathways for COVID-19– positive patients; those with suspected COVID-19 should be isolated until two swabs are completed  Divide Neurovascular Unit into two environments  Contact Minimization: Caretakers and patients wear PPE  Approach to COVID-19–positive patients: Suspected COVID-19: nasal and oropharyngeal swabs. If patient is COVID-19–positive, will be treated in protected area |
| Indian Stroke Association [17] | Consensus Statement – Suggested Recommendations for Acute Stroke Management during the COVID-19 Pandemic: Expert Group on Behalf of the Indian Stroke Association | April 14, 2020 | Imaging: Consider separate CT machine for acute imaging of suspected or positive patients with COVID-19  Thrombolysis/EVT: Standard acute stroke treatment guidelines will apply. Individualized decisions will need to be taken for patients with serious COVID-19 illness  Hospitalization: All COVID-19 suspect stroke cases should be managed in designated COVID-19 health facilities  Contact hospital may consider managing the patient if all guidelines are in place then transferred to COVID hospital  Contact Minimization: Caretakers and patients wear PPE  Early discharge of stable patients with COVID-19  Telemedicine consultation for follow-up visits  Approach to COVID-19–positive patients: Treat suspected or confirmed patients with COVID-19 in designated hospital areas only |
| Society of Neuro-Interventional Surgery [21] | Society of Neuro-Interventional Surgery Recommendations for the Care of Emergent Neuro-interventional Patients in the Setting of COVID-19 | April 15, 2020 | Thrombolysis/EVT: Use guidelines for identification and management of LVO whenever possible  COVID-19–positive: Low threshold for intubation of EVT prior to transport  Unknown COVID-19 status: Treated as high risk, intubation prior to transportation to the angiography suite considered, especially in patients with risk factors  Hospitalization: Transfer uncomplicated postthrombectomy patients out of the ICU^l^ as soon as possible  Recover appropriate EVT patients in non-ICU settings (progressive care/stepdown)  Contact Minimization: COVID-19–negative: standard PPE  Screening for fever and respiratory symptoms  Approach to COVID-19 positivity: Standard institutional protocols  All providers should wear enhanced PPE at all times, provided resources are available.  Patients should be extubated in an isolation ICU room for planned extubation with airborne and contact precautions |
| Recommendations from Rush University Medical Center and Loyola University Medical Center, Chicago [14] | Acute Stroke Care in the Coronavirus Disease 2019 Pandemic | April 17, 2020 | Screening: EMS phone screen for COVID-19 symptoms  Screened for COVID-19 in ED prior to evaluation by the stroke team  Imaging: Diagnostic testing should be consolidated and only be ordered if necessary to initiate appropriate management  Acute imaging at spoke and reviewed both locally and by a telestroke physician  Care Coordination: Patients should be screened for COVID-19 at the spoke site  Thrombolysis/EVT: Thrombolysis patients monitored virtually Patients with large strokes or who require intensive monitoring are admitted to the ICU in a designated COVID-19 rule-out part of the unit with remote rounding with two-way conferencing  Treatment for eligible patients should continue to be offered, even if every vital sign assessment cannot occur in the prescribed time interval  Hospitalization: Separate isolated units planned in advance  Contact Minimization: Telemedicine for patients with low suspicion for stroke or mild symptoms with no potential for intervention  All patients treated as suspected COVID-19 with PPE  PPE according to the CDC^m^ and local institution guidelines (the so-called “protected stroke code”)  Select cases (TIA and small lacunar infarct with minimal deficits) receive expedited testing and critical evaluations in the ED  Telemedicine should be used when possible during rounds  Approach to COVID-19–positive patients: Treatment in a designated separate location |
| Multidisciplinary Group of the Ictus Madrid Plan [12] | Acute Stroke Care During the COVID-19 Pandemic. Ictus Madrid Recommendations | April 21, 2020 | Screening: Patient presumed positive if they have symptoms, live with a person with COVID-19, or lack data  Care Coordination: Ensure ED staff are aware of stroke treatment protocols  Telestroke system to ensure early diagnosis and treatment (including thrombolysis) and avoid unnecessary transfers wherever possible  Contact Minimization: Avoidance of unnecessary procedures  Safe, early discharge when permitted  Telemedicine follow-ups  Approach to COVID-19–positive patients: Protocols developed to minimize treatment delays in possible patients with COVID-19 to ensure isolation and to avoid secondary transfers  Outreach: Awareness campaigns about seeking care in the event of stroke symptoms |
| UCSD^n^ Stroke Center (two academic, certified Comprehensive Stroke Centers) [18] | A Stroke Care Model at an Academic, Comprehensive Stroke Center During the 2020 COVID-19 Pandemic | April 29, 2020 | Screening: Rapid screen for COVID-19; team notified  Imaging: Technicians informed of COVID-19 status and appropriate cleaning measures performed  Care Coordination: ID Stroke Provider First Responder (designated in rotation between faculty providers, advanced practice providers, residents, and vascular neurology fellows)  Thrombolysis/EVT: Treatment proceeds per American Stroke Association and internal hospital guidelines  Pharmacist mixes drugs, but drugs are administered by the stroke team  Angio-suite requires disinfecting via Environmental Health Services for a duration of >30 min  Contact Minimization: Initial consult via telemedicine or telephone with bedside provider  Surgical mask and gloves are used with screen-negative patients  Provider maintains a 6-foot distance except during necessary physical examination for acute stroke  Daily inpatient stroke rounds on hospitalized stroke patients are virtual  Approach to COVID-19–positive patients: Patient with no medical history is treated as screen-positive  Telestroke cases are transferred via the Brain Emergency Management Initiative rapid transfer protocol and treated as screen-positive for COVID-19 |
| National Stroke Workflow Steering Committee [19] | Malaysia Stroke Council Guide on Acute Stroke Care Service During COVID-19 Pandemic | May 3, 2020 | Imaging: Performed in the same setting (CT + CTA and CTP)  CT thorax is not recommended during stroke to diagnose COVID-19  Care Coordination: Minimize number of personnel involved during the stroke activation  Admission of stroke patients to the ICU should be reviewed with attending consultants to provide fair treatment  Thrombolysis/EVT: Do not recommend using single bolus intravenous tenecteplase to minimize exposure to patient  EVT should be performed in a negative pressure room if available  Full PPE, local sedation, or conscious sedation is preferred, and intubation should be avoided in patients who are able to cooperate during the procedure  Hospitalization: Stroke care unit should be maintained to provide evidence-based treatment  Contact Minimization: PPE according to local guidelines for code stroke  Telemedicine and apps are encouraged for consultations  Patients who are stable should wear a surgical face mask at all times  Development of tele-rehabilitation programs  Outreach: Community awareness program for understanding of stroke as a medical emergency during this pandemic |
| International panel of authors [23,28] | Management of acute ischemic  stroke in patients with COVID-19 infection: Report of an international panel  Management of Acute Ischemic Stroke in Patients with COVID-19 Infection: Insights from an International Panel | May 6, 2020 | Imaging: Negative carrier isolator bag may ensure the safety of health care providers  Concurrent pulmonary imaging can identify COVID-19 pulmonary infection  Hospitalization: Policy to identify the principles of decontamination and disinfectants for surfaces in angio-suite  Negative-pressure rooms with anterooms for patients with airborne viral diseases is recommended with or without portable, industrial-grade high efficiency particulate air filter units  Contact Minimization: Maintain a 1-meter distance from patient (unless absolutely necessary) and use PPE  Stroke team should comprise the minimum number of medical professionals and exclude those at high risk  All acute management can be performed via telestroke  Approach to COVID-19–positive patients: Thrombolysis: detailed coagulation profile is preferable beforehand  EVT: intubation, mechanical ventilation, and general anesthesia may be required in a relatively large proportion of cases (if not all cases) |
| Saudi Stroke Society in collaboration with the Saudi Patient Safety Center [26] | Stroke Management Pathway During COVID-19 Pandemic Scientific Statement | June 8, 2020 | Screening: Suspected stroke patients screened for COVID-19 per the Ministry of Health–approved triage for acute respiratory symptoms score  Care Coordination: Field evaluation should be limited to one EMS person  One physician and one nurse can care for the patient  Contact Minimization: Paramedics: full PPE for suspected COVID-19 cases  When feasible, the hospital should have a designated COVID-19 angiography room  Limit admission to selected stroke cases and expedite outpatient stroke services  Telestroke services encouraged to minimize interhospital transfer of stroke patients |
| AHA/ASA^o^ Stroke Council Science Subcommittees: Emergency Neurovascular Care, Telestroke and the Neurovascular Intervention Committees; and on behalf of the Stroke Nursing Science Subcommittee of the AHA/ASA Cardiovascular and Stroke Nursing Council [27] | Pandemic Guidance for Stroke Centers Aiding COVID-19 Treatment Teams | June 23, 2020 | Care Coordination: Reassignment of stroke team to treat COVID-19 with educational resources and centralized clinical guidance to all providers working with patients with COVID-19  Staffing ratios should be flexed to accommodate the important nursing role of emotional support when visitors are limited  In the event of a surge, programmed planning to pull nurses from other service lines or other sites within a health system  Research infrastructure redeployed to rapidly support treatment trials based on the current priority  Hospitalization: Stroke center beds could be converted into COVID-19 units; overflow beds and equipment should be repurposed with flexibility to add or reduce  Contact Minimization: Decrease use of ICU beds and alter protocols to optimize patient care and staff safety  Nursing leadership to identify specific patient populations for stepdown unit or general floor while ensuring patient safety  Telestroke technology platforms could be used by other specialties wanting to perform remote consultations and facilitate telecritical care |

^a^EVT: endovascular therapy.

^b^PPE: personal protective equipment.

^c^MRI: magnetic resonance imaging.

^d^CTA: computed tomography angiography.

^e^TIA: transient ischemic stroke.

^f^CT: computed tomography.

^g^CTP: computed tomography perfusion.

^h^ED: emergency department.

^i^NIHSS: National Institutes of Health Stroke Scale.

^j^EMS: emergency medical services.

^k^FAST: Functional Assessment Staging Test.

^l^ICU: intensive care unit.

^m^CDC: US Centers for Disease Control and Prevention.

^n^UCSD: University of California San Diego.

^o^AHA/ASA: American Heart Association/American Stroke Association.

Table S2. Key studies evaluating acute stroke trends during the COVID-19 pandemic.

| Study title (publication  date) | Primary aim of study | Location of study | Study type and enrollment | Eligibility criteria | Key findings |
| --- | --- | --- | --- | --- | --- |
| Collateral Effect of COVID-19 on Stroke Evaluation in the United States [44] (May 8, 2020) | To quantify the quantity of care that hospitals provided to patients with acute ischemic stroke | 856 hospitals in the United States | Retrospective record analysis of imaging data using RAPID software; 231,753 total enrolled | Dates: July 1, 2019, through April 27, 2020  Criteria: Patients who underwent imaging processed with RAPID software  Procedure: RAPID software was used to process any of the following types of imaging: non-contrast CT^a^ or MRI^b^ of the brain, CT or MR^c^ perfusion of the brain, or CT or MR angiography of the head and neck | - Imaging numbers decreased by 39%, from 1.18 patients per day per hospital prepandemic to 0.72 patients per day per hospital early in the pandemic - Texas had a mean of 63.3 patients per day (95% CI 60.2-66.5) prepandemic and 43.1 patients per day (95% CI 40.1-46.2) early in the pandemic, with a relative reduction of 31.8% (95% CI –25.8% to 37.6%) - Decrease of approximately 39% in the numbers of patients who received evaluations for acute stroke between two recent epochs in US hospitals |
| Falling Stroke Rates During COVID-19 Pandemic at a Comprehensive Stroke Center [45] (May 14, 2020) | To estimate the change in the number of new stroke diagnoses in the region | Comprehensive stroke center in New Jersey (that serves as a referral base for 5 primary stroke centers) | Exploratory single-center retrospective analysis of prospective cohort. Total of 328 patients enrolled; 53 patients (16%) with COVID-19 | Dates:   - Pre–COVID-19: October 1, 2019, to February 29, 2020 - COVID-19: March 1 to April 15, 2020   Criteria:   - Adults aged >18 years - Final diagnosis of acute ischemic stroke | - Decrease in new stroke diagnoses by 38% (mean 1.82 per day, SD 1.38, to mean 1.13 per day, SD 1.07); *P*<.01 - No significant change in the monthly number of strokes due to LVO^d^ - Increased proportion of new LVOs in the COVID-19 period (38% vs 21%; *P*=.01) - Higher odds of LVO remained significant after adjustment for age and stroke severity (aOR^e^ 2.06, 95% CI 1.02- 4.16; *P*=.04) - Fewer brain MRIs were performed during the COVID-19 period vs pre–COVID-19 (mean 148 per month vs 349 per month, *P*<.01) - No significant delay from TLKW^f^ to ED^g^ arrival, to CT scan, or to thrombolysis - Shorter hospital length of stay during COVID-19 vs pre-COVID-19 (𝛃=–2.91, 95% CI –5.83 to 0.02; *P*=.05) - Remained significant after adjustment for age, NIHSS^h^ score, and presence of LVO (adjusted 𝛃=–3.39, 95% CI –6.10 to –0.68, *P*=.01) - No significant differences with respect to age, sex, race, vascular risk factors, or stroke severity |
| Acute Stroke Care in the Era of COVID-19 (Barcelona) [30] (May 22, 2020) | To analyze how the COVID-19 pandemic affected acute stroke care | Network of 26 acute hospitals in Catalonia | Single-center, observational cohort study. Total of 943 stroke codes (n=517 in 2019 and n=426 in 2020); 191 total stroke admissions (n=108 in 2019 and n=83 in 2020) | Dates: March 2019 compared to March 2020  Procedure: Measured the number of emergency calls and stroke codes to the Emergency Medical System in Catalonia | - Relative to March 2019, the Emergency Medical System had a 330% mean increment in the number of calls (158,005 vs 679,569) - Fewer stroke code activations (517 vs 426) - Fewer stroke admissions (108 vs 83) and thrombectomies (21 versus 16) - Younger age was found in stroke admissions during the pandemic (median 69 years, IQR 64-73, vs median 75 years, IQR 73-80; *P*=.009) |
| Delayed Presentation of Acute Ischemic Strokes During the COVID-19 Crisis [58] (May 28, 2020) | To evaluate delay in presentation for acute ischemic stroke patients in the first month of the COVID-19 pandemic in the United States | 12 stroke centers across the United States | Retrospective review of prospective ongoing data collection. 10 total patients analyzed. Baseline: February to March 2019 (n=320); pre–COVID-19: February 2020 (n=227); COVID-19: March 2020 (n=163). | Criteria: acute ischemic stroke as adjudicated by the GTWG^i^ database entry of the final diagnosis  Dates:   - Baseline: February to March 2019 - Pre–COVID-19: February 2020 - COVID-19: March 2020 | - No significant difference in the severity of presentation; mean initial NIHSS scores were 9.2, 9.7, and 9.6 for the baseline, pre–COVID-19, and COVID-19 periods, respectively (*P*=.79) - Increased mean TLKW and presentation was 442±435 minutes for the baseline period, 552±630 minutes for the pre–COVID-19 period, and 603±1035 minutes for the COVID period (163-minute mean increase; *P*<.03) - For patients with NIHSS scores >4: increased mean TLKW to presentation was 405±50 minutes for the baseline period and 613±70 minutes for the COVID-19 period, remaining significant (*P*<.02) |
| Under-utilization of Health Care for Strokes During the COVID-19 Outbreak [46] (June 1, 2020) | To investigate the decrease in stroke cases presenting to the ED | Global clinical research platform | Retrospective analysis of clinical research databases. 155,156 total cases; Control 1 (n=66,671); Control 2 (n=50,921); COVID-19 (n=37,941). | Criteria: Ischemic stroke patients in the TriNetX database >18 years of age  Dates:   - COVID-19: January 20 to May 16, 2020 - Control: January 20 to May 16, 2019 - Control: September 25, 2019, to January 19, 2020 | - Decreases in stroke patients of 43% compared to control 1 and 25.5% compared to control 2 - Decreases in number of IV^j^ tPA^k^ administrations by 51% and 35% compared to control 1 and control 2, respectively |
| Morbidity and Mortality Weekly Report - Impact of COVID on ED Visits [54] (June 3, 2020) | To quantify the effects of COVID-19 on US ED visits | 47 states (all but Hawaii, South Dakota, and Wyoming) | The CDC^l^ compared the volume of ED visits during four weeks early in the pandemic. Data were obtained from the National Syndromic Surveillance Program on the number of ED visits broken down by age and region for the 47 states under study (estimated 3552 ED visits reported in the most recent week) | Dates:   - Control: March 31 to April 27, 2019 - Early COVID-19: March 29 to April 25, 2020 | - Decreased ED visits by 42% compared to 2019 - Largest declines in ED visits by persons who were aged ≤14 years, female, and in the northeast region |
| COVID-19 Pandemic—The Bystander Effect on Stroke Care in Michigan [47] (June 4, 2020) | To describe the bystander effect of the COVID-19 pandemic on ischemic and hemorrhagic stroke | 11 comprehensive stroke centers and 1 primary stroke center in Michigan and northwest Ohio | Retrospective analysis. Total enrollment of 1760: 518 cases in March 2020 (incidence rate: 16.71), 610 in the February 2020 control group (incidence rate: 21.03), and 632 in the March 2019 control group (incidence rate: 20.39) | Criteria: 11 comprehensive stroke centers and 1 primary stroke center  Dates:   - Control 1: February 2020 - Control 2: March 2019 - COVID-19: March 2020 | - Decreased rate of ischemic stroke admissions for March 2020 vs February 2020 (IRR^m^ 0.85, 95% CI 0.76-0.95; *P*=.006) and March 2019 (IRR: 0.82, 95% CI 0.73-0.92; *P*=.001). - Decreased rate of EVT for March 2020 vs February 2020 (IRR 0.57, 95% CI 0.40-0.81; *P*=.002) and March 2019 (IRR 0.61, 95% CI 0.43-0.88; *P*=.007). - No significant changes in baseline characteristics; particularly, no significant difference in the time from onset to presentation or length of hospital stay - No significant difference in the administration of tPA between the study time period and controls |
| Acute Stroke in Times of the COVID-19 Pandemic: A Multicenter Study [31] (June 9, 2020) | To assess the number of patients seeking inpatient emergency care for AIS^n^ during the COVID-19 pandemic | 4 German academic stroke centers | Retrospective multicenter study | Criteria: Patients admitted with final diagnosis of ischemic stroke or TIA^o^  Dates:   - Control: January 1 to April 14, 2019 - COVID-19: January 1 to April 12, 2020 | - Decrease in number of TIA admissions (−85%, −46%, −42%) in 3 of 4 centers - Stroke admission rates decreased significantly by 40% and 46% after week 12 in 2020 in 2 of 4 centers |
| Decrease in Hospital Admissions for Transient Ischemic Attack, Mild, and Moderate Stroke During the COVID-19 Era [32] (June 12, 2020) | To investigate the impact of the onset of the COVID-19 pandemic on stroke admissions | 6 hospitals in Joinville, Brazil | Retrospective analysis of a population-based stroke registry; 1169 patients with stroke, 917 admitted | Criteria: Patients included in the population-based Joinville Stroke Registry (Joinvasc)  Dates: January 2019 to April 15, 2020 | - Decreased total stroke admissions (–36.4%) from an average of 12.9/100,000 per month in 2019 to 8.3 after COVID-19 (*P*=.003) - No difference in admissions for severe stroke (NIHSS >8), intraparenchymal hemorrhage, or subarachnoid hemorrhage |
| Letter to the Editor: Incidence of Acute Ischemic Stroke and Rate of Mechanical Thrombectomy During the COVID-19 Pandemic in a Large Tertiary Care Telemedicine Network [48] (June 13, 2020) | To improve the understanding of the effect of the COVID-19 pandemic on stroke frequency and care | Tertiary telestroke network in Philadelphia, PA | Retrospective analysis of the incidence of acute ischemic stroke and EVT^p^. 496 telestroke consultations; 257 acute ischemic stroke admissions; 69 mechanical thrombectomies | Dates:   - COVID-19: March 15 to April 30, 2020 - Control: 2017, 2018, 2019 | - Decreased AIS admissions by 23% (*P*=.001) and telestroke consultations by 48% (*P*=.001) compared with previous years - Increased number of EVTs by 50% (*P*=.11) during COVID-19 - 25% of all EVT patients tested positive for COVID-19, which was significantly higher than the expected frequency (incidence of COVID-19 is 1.1%) - No significant delay in diagnosis to intervention (493 minutes vs 544 minutes, *P*=.40) |
| Decline in Stroke Presentations During  COVID-19 Surge [49] (June 18, 2020) | To investigate acute stroke presentations during the COVID-19 pandemic | 19 emergency departments in northeast Ohio | Retrospective analysis of stroke registry; 902 total stroke alerts | Dates:   - Control: January 1 to March 8, 2020 - COVID-19: March 9 to April 2, 2020 | - Decreased stroke alerts (–20%) from median 10 (IQR 8-13) during baseline to median 8 (IQR 4-10) during COVID-19 (*P*=.001) - No change in time to presentation, stroke severity, or time to treatment - Decreased thrombolysis with a rate ratio of 0.52 (95% CI 0.28-0.97) - No change in EVT (rate ratio 0.93, 95% CI 0.52-1.62) |
| Potential Indirect Effects of the COVID-19 Pandemic on Use of Emergency Departments in US Jan-May 2020 [55] (June 22, 2020) | To describe trends in ED numbers for myocardial infarction, acute stroke, and hyperglycemic events | 47 states (all but Hawaii, South Dakota, and Wyoming) | Retrospective analysis of the National Syndromic Surveillance Program to assess trends in ED visits | Dates:   - Pre–COVID-19: January 5 to March 14, 2020 - COVID-19: March 15 to May 23, 2020 | - ED visits declined by 23% for myocardial infarction, 20% for stroke (57,490 vs 46,066), and 10% for hyperglycemic crisis compared with prior to the pandemic (similar for male/female patients) - Absolute decrease in ED visits for stroke was largest among men aged 65 to 74 years (1406-visit decrease) and women aged 75 to 84 years (1642-visit decrease) - Larger decreases were seen in older women |
| Acute Stroke Management During the COVID-19 Pandemic: Does Confinement Impact Eligibility for Endovascular Therapy? [33] (July 1, 2020) | To determine the effects on EVT for patients with AIS during COVID-19 confinement | 17 stroke centers (6 were based in France, 3 in Portugal, 2 in Italy, 2 in Switzerland, 1 in Spain, 1 in Germany, 1 in Canada, and 1 in the United States) | Retrospective observational study. Total enrollment 1600; onset-to-groin puncture time (n=776) | Dates: November 1, 2019, to April 15, 2020 | - Decreased mean number of EVTs/hospital per 2-week interval (–32%) from before COVID-19 (mean 9.0, 95% CI 7.8-10.1) to during COVID-19 (mean 6.1, 95% CI 4.5-7.7; *P*<.001) - Increased TLKW to groin puncture (EVT) by 54 minutes from before COVID-19 (mean 300.3 minutes, 95% CI 285.3-315.4) to during COVID-19 (mean 354.5 minutes, 95% CI 316.2-392.7; *P*<.001) |
| Effect of COVID-19 pandemic on stroke admission rates in a Norwegian population [34] (July 3, 2020) | To evaluate how lockdown due to the pandemic affected the number of admissions of acute stroke | Akershus University Hospital, Norway | Retrospective hospital chart review. Total of 323 enrolled: pre–COVID-19 (n=218); COVID-19 (n=105) | Criteria: discharged with a diagnosis of TIA or acute stroke  Dates:   - Pre–COVID-19: January 3 to March 12, 2020 - COVID-19: March 13 to April 30, 2020 | - Decreased weekly stroke admissions (mean 21.8, SD 4.7, range 29-14, before the lockdown and mean 15.0, SD 4.2, range 21-8, during COVID-19; *t* test, *P*=.008) - Increased severity (average NIHSS at admission) during COVID-19 vs before lockdown (5.9 vs 4.2; *t* test, *P*=.04) - Proportion of patients with ischemic stroke reaching hospital within 4.5 hours (thrombolysis) was significantly higher before than during COVID-19 (45% vs 30%; chi-square test, *P*=.03) |
| Characteristics and Outcomes in Patients With  COVID-19 and Acute Ischemic Stroke [35] (July 9, 2020) | To assess whether stroke severity (estimated by the NIHSS) and outcomes (assessed by the mRS^q^ at discharge) in patients with AIS are different before vs during COVID-19 | Multicenter international registry, Lausanne, Switzerland | Retrospective analysis of registry using 1:1 propensity score matching. Total enrollment: 330: COVID-19 (n=165); COVID-19 and AIS (n=174) | Criteria:   - Patients hospitalized with laboratory-confirmed COVID-19 and AIS - Excluded patients who were infected after the onset of stroke   Dates:   - COVID-19: January 27 to May 19, 2020 - Control: 2003 and 2019 | - Increased risk of severe disability for patients with COVID-19 (median mRS 4, IQR 2-6) compared with non–COVID-19 patients (median mRS 2, IQR 1-4; *P*<.001 - Increased risk of death for patients with COVID-19 (OR^r^ 4.3, 95% CI 2.22-8.30) compared with non–COVID-19 patients - No difference in the prevalence of large-artery and lacunar stroke between the 2 populations (chi square test, *P*=.082) |
| Impact of the COVID-19 pandemic on hyperacute stroke treatment (Singapore) [36] (July 13, 2020) | To study the overall volume of AIS cases and the delivery of hyperacute stroke services during DORSCON^s^ Orange | Comprehensive stroke center in western region of Singapore | Single-center, observational cohort study. Total enrollment 350: pre–COVID-19 (n=206, 58.9%); COVID-19 (n=144, 41.1%) | Criteria: patients reviewed as an acute stroke activation in the ED  Dates: November 2019 to April 2020 | - Decreased stroke codes during COVID-19 (144) vs pre–COVID-19 (206) (95% CI 6.513-2.287; *P*=.004) - No change in proportion of stroke codes receiving acute recanalization therapy pre–COVID-19 (28.6%) vs during COVID-19 (32.6%; *P*=.42) |
| The impact of the COVID-19 pandemic on  a stroke center in Latin America [37] (July 14, 2020) | To share the impact of the COVID-19 pandemic on stroke care in Latin America | Comprehensive stroke center in Niteroi, Rio de Janeiro, Brazil | Retrospective analysis of hospital data. Total analyzed=70: baseline (n=45); COVID-19 (n=25) | Dates:   - March to May 2019 - March to May 2020 | - Decreased stroke admissions of 44.5% in the COVID-19 period (45 cases) compared to the previous year (25 cases) - Decrease of 41.7% in ischemic stokes during COVID-19 - No difference in quality of care metrics or severity of stroke was observed during the pandemic |
| Impact of the COVID-19 Outbreak on Acute Stroke Care [51] (July 20, 2020) | To assess the impact of the COVID-19 outbreak on trends in hospital admissions and workflow parameters | 3 hospitals in Amsterdam, the Netherlands | Retrospective multicenter cohort study of prospective stroke registries. Total patients enrolled=716:COVID-19 (n=309); pre–COVID-19 (n=407). | Criteria: consecutive patients presenting to the emergency departments with suspected stroke or code stroke  Dates:   - COVID-19: March 16 to May 3, 2020 - Pre–COVID-19: October 21 to December 8, 2019 | - Decreased suspected stroke presentations (–24%) during the COVID-19 outbreak (n=309) compared to pre–COVID-19 (n=407), IRR 0.76 (95% CI 0.65-0.88) - No change in the proportion of stroke patients treated with intravenous thrombolysis (28% vs. 30%, *P*=.58) - No change in the proportion of EVT (11% vs 12%, *P*=.82) - No change in onset-to-door time (187 minutes vs 150 minutes, *P*=.39) - Proportion of men was higher during the COVID-19 period (59% vs 47%, *P*<.001) - No change in NIHSS score (4 vs 4, *P*=.55) or proportion of large vessel occlusions (26% vs 22%, *P*=.44) |
| Impact of the Coronavirus Disease Pandemic on the Number of Strokes and Mechanical Thrombectomies: A Systematic Review and Meta-Analysis [53] (July 22, 2020) | To evaluate the impact of COVID-19 on the number of stroke alerts/codes, number of reperfusions, and number of thrombectomies | N/A^t^ | Systematic review and meta-analysis of 59,233 strokes from 9 studies | Criteria: Original articles, case studies, research letters, short reports containing primary data | - Decreased number of stroke alerts during COVID-19, 64% (56%-71%) of that during the pre-pandemic period - Decreased number of reperfusion therapies during COVID-19, 69% (61%-77%) of that during the pre-pandemic period - Decreased number of EVTs during COVID-19; pooled analysis showed that the number of EVTs during the pandemic was 78% (75%-80%) of that during the prepandemic period - Increased incidence of EVTs during COVID-19 (OR 1.23, 95% CI 1.12-1.36, *P*<.001; I^2^=0%, *P*=.85) |
| Stroke Code Presentations, Interventions, and Outcomes Before and During the COVID-19 Pandemic [52] (July 31, 2020) | To quantify trends in stroke code calls and treatments | 3 Connecticut hospitals | Retrospective analysis of stroke code activity. Total of 822 stroke codes enrolled: control (n=786); COVID-19 (n=756). | Dates:   - COVID-19: January 1 to April 28, 2020 - Control: January 1 to April 28, 2019 | - Decreased weekly stroke code volumes from January to April 2020 (n=756) compared with 2019 (n=786), with 2.5 fewer stroke codes called each week (*P*<.001, *R*^2^=0.7163). - Increased likelihood of the following risk factors during COVID-19 (n=211) compared to prepandemic (n=167): hypertension (*P*=.04), hyperlipidemia (*P*=.02), coronary artery disease (*P*=.04), or substance abuse (*P*=.01), less likely to have private health insurance (*P*=.046), and more likely to live in the CSC^u^ city (*P*<.001) - Decreased total stroke codes during COVID-19 (from week 8 onwards) by 30.0% compared with the corresponding weeks in 2019, piecewise linear regression slope of −12.8 calls per week from weeks 8 to 11 (*P*=.03, *R*^2^=0.9416) - No significant differences in sex, age, race/ethnicity, premorbid mRS, prestroke antiplatelet or anticoagulation use, histories of diabetes, atrial fibrillation, prior stroke or transient ischemic attack, congestive heart failure, chronic obstructive pulmonary disease, malignancy, or tobacco use |
| Impact of the COVID-19 Epidemic on Stroke Care and Potential Solutions [56] (July 31, 2020) | To investigate the impact of the COVID-19 outbreak on stroke care | 280 hospitals across China in 29/31 provinces and municipalities | Retrospective analysis of Big Data Observatory Platform for Stroke of China; 227 patients enrolled | Dates:   - COVID-19: February 2020 - Control: February 2019 | - Decreased total number of thrombolysis cases (–26.7%) in February 2020 (n=2508) compared to February 2019 (n=3422), *P*<.001 - Decreased total number of thrombectomy cases (–25.3%) in February 2020 (n=970) compared to February 2019 (n=1298), *P*<.001 |
| Blacks Are Less Likely to Present With Strokes During the COVID-19 Pandemic: Observations From the Buckle of the Stroke Belt [65] (August 5, 2020) | To investigate trends in telestroke consultations during the pandemic | 27 telestroke hospitals in South Carolina | Retrospective chart review of consecutive telestroke patients. Total enrollment 5852: COVID-19 (n=613, 10.5%) | Criteria: Consecutive patients seen through telestroke  Dates:   - Pre–COVID-19: March 2019 to February 2020 - COVID-19: March to April 2020 | - Decreased median number of weekly consultations from 112 to 77 during the pandemic, *P*=.002 - No difference in baseline features (age, sex, or time to presentation) - Black patients less likely to present with strokes during COVID-19 (85/615, 13.9%, vs 1519/5852, 29%; *P*≤.002) - Decreased number of EVTs per week during COVID-19 (1, IQR 0.5-1, versus 3, IQR 1-4; *P*=.02), but similar percentages received EVT - No change in treatment time - Increased percentage of patients receiving intravenous tPA during the pandemic (15.5% vs 12.5%, *P*=.04) |
| Acute Stroke Presentation, Care, and Outcomes in Community Hospitals in Northern California During the COVID-19 Pandemic [50] (August 7, 2020) | To compare temporal trends in volume of acute stroke alerts, patient characteristics, telestroke care, and short-term outcomes | 21 stroke centers in Northern California | Retrospective cohort study. Total enrollment 9120: pre–COVID-19 (n=8337); COVID-19 (n=783) | Criteria: Adult patients presenting with suspected acute stroke and evaluated by telestroke  Dates:   - Pre–COVID-19: January 1, 2019, to March 14, 2020 - COVID-19: March 15 to May 9, 2020 | - Decreased stroke alert weekly volume during COVID-19 (mean 98, 95% CI 92-104) compared with pre–COVID-19 (mean 132, 95% CI 130-136; *P*<.001) - No differences in demographics - Increased severity during COVID-19 (median NIHSS score 8 vs 9, *P*=.003) - Increased proportion of LVO during COVID-19 (64/363, 17.6%, compared with pre–COVID-19 (466/3474, 13.4%; *P*=.03) |
| Low stroke incidence in the TEMPiS^v^ telestroke network during COVID-19 pandemic – effect of lockdown on thrombolysis and thrombectomy [57] (August 18, 2020) | To evaluate the effects of the COVID-19 lockdown on stroke consultations and treatment recommendations | 12 clinics without neurology departments in the telestroke network TEMPiS (Bavaria, Germany) | Retrospective analysis of the Bavarian telestroke database TEMPiS; 7608 acute telemedical consultations | Dates:   - Pre–COVID-19: January to April 2017, 2018, and 2019 - COVID-19: January to April 2020 | - Decreased IV thrombolysis recommendation (23/250, 9.2%, in 2020 versus 148/1006, 14.7%) in consultations between 2017 and 2019, *P*=.02 - No difference in EVT recommendations for the time period between March 16 and April 30 in 2020; 19/250 (7.6%) compared with the same time periods in 2017, 2018, and 2019 (74/1006, 7.4%) |
| Early Brain Imaging Shows Increased Severity of Acute Ischemic Strokes With Large Vessel Occlusion in COVID-19 Patients [66] (August 19, 2020) | To compare anterior circulation LVO stroke severity between patients with and without COVID-19 | Paris, France | Comparative cohort study. Total enrollment 46: COVID-19 (n=12); control (n=34) | Criteria:   - Anterior LVO - Imaging ≤3 hours from onset - PCR^w^-confirmed COVID-19   Dates:   - March 15 to April 30, 2020 - Controls presenting March 15 to April 30, 2019 | - Patients with COVID-19 were younger; no differences in time from onset or NIHSS - Patients with COVID-19 had more severe strokes on imaging than patients without COVID-19, with a significantly lower clot burden score (median: 6.5 versus 8, *P*=.016), higher rate of multivessel occlusion (50% versus 8.8%, *P*=.005), lower DWI-ASPECTS^x^ (median 5 versus 8, *P*=.006), and higher infarct core volume (median 58 versus 6 mL, *P*=.004) - • In-hospital mortality was higher in the group of patients with COVID-19 (41.7% versus 11.8%, *P*=.025). |

^a^CT: computed tomography.

^b^MRI: magnetic resonance imaging.

^c^MR: magnetic resonance.

^d^LVO: large vessel occlusion.

^e^aOR: adjusted odds ratio.

^f^TLKW: time last known well.

^g^ED: emergency department.

^h^NIHSS: National Institutes of Health Stroke Scale.

^i^GTWG: Get With The Guidelines.

^j^IV: intravenous.

^k^tPA: alteplase.

^l^CDC: US Centers for Disease Control and Prevention.

^m^IRR: incidence rate ratio.

^n^AIS: acute ischemic stroke.

^o^TIA: transient ischemic attack.

^p^EVT: endovascular treatment.

^q^mRS: modified Rankin score.

^r^OR: odds ratio

^s^DORSCON: Disease Outbreak Response System Condition

^t^N/A: not applicable.

^u^CSC: Comprehensive Stroke Center.

^v^TEMPiS: TeleMedical Project for integrative Stroke Care.

^w^PCR: polymerase chain reaction.

^x^DWI-ASPECTS: Diffusion-Weighted Imaging–Alberta Stroke Program Early Computed Tomography Scores.
